# Establishment and Application of an Indirect ELISA for Detecting Getah Virus IgG Antibodies in Swine Based on the E2EP3 Peptide

**DOI:** 10.3390/vetsci13060530

**Published:** 2026-05-29

**Authors:** Sihao Peng, Rongrong Li, Yuxin Yang, Xin An, Xi Zhu, Ruidong Li, Yuanyuan Liu, Rui Wu, Qi-Gui Yan, Yiping Wen, San-Jie Cao, Xiaobo Huang, Qin Zhao, Yiping Wang, Yi-Fei Lang, Shan Zhao, Fei Zhao, Yi Zheng, Jinxin Meng, Lu Chen, Senyan Du

**Affiliations:** 1 Research Center for Swine Diseases, College of Veterinary Medicine, Sichuan Agricultural University, Chengdu 611130, China; 18884737596@163.com (S.P.); 15682556823@163.com (R.L.); yyx2277@163.com (Y.Y.); anxin29292x@163.com (X.A.); 15283276421@163.com (X.Z.); liruidong764@163.com (R.L.); 18373980318@163.com (Y.L.);; 2 Sichuan Science-Observation Experimental Station for Veterinary Drugs and Veterinary Diagnostic Technology, Ministry of Agriculture, Chengdu 611130, China; 3 Engineering Research Center of Southwest Animal Disease Prevention and Control Technology, Ministry of Education of the People’s Republic of China, Chengdu 611130, China; 4 Yunnan Veterinary and Animal Science Institute, Kunming 650224, China; 5 College of Veterinary Medicine, Henan Agricultural University, Zhengzhou 450046, China

**Keywords:** Getah virus, ELISA, E2EP3, antibody detection

## Abstract

The Getah virus (GETV) is a mosquito-borne pathogen that infects diverse hosts, including pigs, horses, and humans, which can cause swine reproductive disorders such as abortion and stillbirth, posing a potential threat to animal and public health. Therefore, there is an urgent need for efficient and accurate serological diagnostic methods for surveillance and control of GETV. However, commercial diagnostic kits for swine GETV infection remain unavailable. In this study, we developed a novel enzyme-linked immunosorbent assay (ELISA) based on a GETV-specific epitope peptide (E2EP3) for serological detection. The N-terminally biotinylated E2EP3 peptide was synthesized, and the reaction conditions were systematically optimized, resulting in a cut-off value of 0.363. The assay exhibited no cross-reactivity with Japanese encephalitis virus (JEV), porcine circovirus type 2 (PCV2), porcine circovirus type 3 (PCV3), pseudorabies virus (PRV), or classical swine fever virus (CSFV). It demonstrated good reproducibility and high sensitivity, detecting GETV-positive serum diluted up to 1:640. The overall agreement rate reached 95%, consistent with a conventional recombinant GETV E2 protein-based ELISA. Benefiting from the biotin–streptavidin system, this assay achieved strong signal amplification and low background. Moreover, the procedure is simple, cost-effective, and stable, making it suitable for GETV large-scale serological surveillance and vaccine evaluation.

## Simple Summary

As a member of the *Alphavirus*, the Getah virus (GETV) is mainly transmitted by mosquito vectors. The virus has a broad host range and poses a serious threat to the pig breeding industry and public health security. However, commercial serological detection kits for porcine Getah virus are still unavailable, which can hardly meet the needs of clinical diagnosis and epidemic prevention and control. In this study, a GETV-specific linear epitope E2EP3 polypeptide was used as the target antigen. The established ELISA showed high sensitivity, specificity, and reproducibility. The results indicated that this ELISA was highly consistent with the conventional E2-ELISA in the detection of clinical samples, providing a practical and cost-effective approach for large-scale serological surveillance of GETV.

## 1. Introduction

The Getah virus (GETV) is a mosquito-borne virus belonging to the genus *Alphavirus* in the family *Togaviridae.* It is considered an emerging zoonotic pathogen with a broad host range, capable of infecting humans and multiple animal species, including pigs, horses, cattle, and farmed fur-bearing animals such as raccoon dogs [1,2,3]. GETV is primarily transmitted by mosquitoes, particularly species of the genus *Culex* [4]. In piglets, GETV infection can cause fever, anorexia, diarrhea, tremors, arthritis, hind limb paralysis, ataxia, and high mortality. In pregnant sows, infection is mainly associated with reproductive disorders, such as abortion and stillbirth [5]. Serological investigations have detected antibodies against GETV in human sera, suggesting that humans may also be exposed to or infected with the virus [6,7].

Since the first GETV strain was isolated from a mosquito in Hainan province, China, in 1964, the virus has been reported in at least 17 provinces across the country [8]. In recent years, outbreaks of GETV infection have been documented on commercial pig farms, including a concentrated epidemic reported in Henan Province between July and September [9], highlighting the increasing prevalence of the virus and its potential threat to the swine industry and public health. In recent years, GETV-associated reproductive disorders have been increasingly reported in pig populations in East and Southeast Asia [10,11]. Currently, there are no commercial vaccines or specific antiviral drugs available for GETV, making rapid and accurate diagnostic methods essential for effective surveillance and outbreak control [12].

Although virus isolation and identification remain the gold standard for confirming viral infection, these methods are labor-intensive, time-consuming, and require specialized laboratory facilities [13]. Molecular diagnostic techniques, such as quantitative real-time PCR, enable sensitive detection of viral nucleic acids but are also relatively time-consuming and require specialized equipment [14,15]. In contrast, serological assays—including virus neutralization testing (VNT), hemagglutination inhibition testing (HI), and enzyme-linked immunosorbent assay (ELISA)—provide practical and cost-effective approaches for detecting virus-specific antibodies and assessing infection status [16,17,18]. Among these methods, ELISA is particularly advantageous due to its high throughput, specificity, sensitivity, and ease of standardization, making it suitable for large-scale surveillance [19,20].

The GETV envelope is composed of heterodimers of two transmembrane glycoproteins, E1 and E2, which are responsible for virus attachment and membrane fusion during viral entry [21,22]. The E2 glycoprotein is located on the virion surface and plays a critical role in inducing host immune responses and neutralizing antibodies against GETV infection [23]. Because of its immunogenicity and relatively conserved sequence, the E2 protein has been widely used as an antigen in serological assays for GETV detection [24]. Previous studies have developed ELISA methods for detecting GETV infection in horses using recombinant E2 protein or synthetic peptides as the antigen [25]. Similarly, ELISA assays based on recombinant E2 protein have been reported for the serological detection of GETV infection in pigs [12,18]. However, recombinant E2 protein is frequently expressed in *Escherichia coli* as inclusion bodies [26,27], which can complicate purification procedures and reduce production efficiency. To overcome these limitations, peptide-based ELISAs have been proposed as an alternative approach because synthetic peptides can be produced with high purity, good batch-to-batch consistency, and relatively simple preparation processes [28]. However, peptide-based ELISA methods targeting well-defined immunodominant epitopes for the serological detection of GETV infection in pigs remain limited, and their diagnostic performance has not been fully evaluated.

E2EP3 is an 18-amino acid peptide located in the N-terminal region of the E2 glycoprotein, and its sequence is highly conserved among most GETV isolates. This peptide has been identified as a dominant B-cell epitope during alphavirus infection. In particular, E2EP3 has been reported as an early serological biomarker in Chikungunya virus (CHIKV), another member of the *alphavirus* genus GETV [29,30]. Previous studies demonstrated that the ELISA system based on the E2EP3 peptide could detect anti-E2EP3-IgG antibodies during the acute phase of CHIKV infection [31,32], showing good sensitivity and potential for early diagnosis.

In the present study, we developed and optimized an ELISA method based on biotinylated E2EP3 peptide for the detection of GETV-specific antibodies. The assay was evaluated using mouse and pig sera to determine its sensitivity, specificity, and reproducibility. This peptide-based ELISA provides an alternative serological tool for the rapid diagnosis and large-scale epidemiological surveillance of GETV infection in pigs, and may contribute to improved prevention and control strategies for GETV.

## 2. Materials and Methods

### 2.1. Virus, Cells, and Sera 

The GETV strain SC202009 (GenBank accession OK423758.1) was preserved at the Swine Disease Center of Sichuan Agricultural University [24]. The virus was propagated in baby hamster kidney 21 (BHK-21) cells using Dulbecco’s Modified Eagle’s Medium (DMEM) supplemented with 2% (*v*/*v*) fetal bovine serum (FBS; Gibco, Beijing, China). Porcine serum samples positive for PCV-2, PCV-3, CSFV, JEV, PRV, PRRSV, and GETV, as well as GETV-negative serum samples, were stored in the laboratory of the Swine Disease Center, Sichuan Agricultural University. All negative porcine sera for cut-off determination were collected from age-matched pigs under uniform rearing conditions. These samples were derived from a GETV-free closed herd and strictly verified as GETV-negative by VNT and PCR. This design eliminated interference from individual variation and environmental factors, guaranteeing the reliability of the diagnostic threshold. The GETV-positive porcine sera used in this study were collected from clinically positive pigs in commercial pig farms of Henan and Yunnan Provinces within the past five years. 

### 2.2. Peptide Synthesis and Animals

The synthesized biotinylated-peptide E2EP3 (SVTKHFNVYKATKPYLAY) was synthesized by GenScript Biotechnology Co., Ltd. (Nanjing, China). Biotinylation modification was performed at the N terminus of the peptide. Ten SPF female Balb/C mice (8 weeks old) were purchased from Beijing Vital River Laboratory Animal Technology Co., Ltd. Mice were housed in a controlled environment with free access to food and water. All experimental procedures were reviewed and approved by the Animal Care and Use Committee of Sichuan Agricultural University (license number: 20250031, Chengdu, China).

### 2.3. Amino Acid Sequences of the E2EP3 Region of the E2 Protein of Getah Virus Strains and Other Alphaviruses

Amino acid sequences of the E2EP3 region of the E2 protein were compared among various Getah virus strains (*n* = 11) and other alphaviruses, including Chikungunya virus (CHIKV), eastern equine encephalitis virus (EEEV), Sindbis virus (SINV), and western equine encephalitis virus (WEEV).

### 2.4. Mouse Sera

To obtain the standard positive serum, 8-week-old Balb/C mice (*n* = 5) were challenged with 5 ×10^5^ PFU of the GETV strain SC202009 by intraplantar injection, and boosted at 1 and 2 weeks after the initial challenge. The other five mice were injected with an equal volume of PBS to serve as the negative control. Blood samples were collected at 3 weeks. After standing at room temperature for 4 h, the blood was centrifuged at 1500× *g* at 4 °C to harvest serum [33,34]. ELISA was performed with 1 μg of E2 protein coated per well to determine antibody titers. Meanwhile, ELISA plates coated with streptavidin and E2EP3 were used to measure the OD450 nm values of pre-immune serum and post-immune serum.

### 2.5. Establishment of Indirect ELISA Method

#### 2.5.1. Optimization of Reaction Conditions

Optimal antigen concentration and serum dilution factor were determined through square array titration. Based on the optimized binding molar ratio of streptavidin to biotinylated peptide (1:200) determined in preliminary experiments, streptavidin was diluted into three gradients (1 μg/mL, 2 μg/mL, and 3 μg/mL) using a coating solution (1× PBS), with 100 μL added to each enzyme labeling well. The plates were incubated overnight at 4 °C. After washing three times with PBST, the E2EP3 peptide was diluted into three gradients (7.5 μg/mL, 15 μg/mL, and 30 μg/mL) using a coating solution (1× PBS), with 100 μL added to each enzyme labeling well. The plates were incubated overnight at 4 °C and subsequently blocked with 5% skim milk at 37 °C for 2 h. Negative and positive serum samples were diluted in four gradients (1:50, 1:100, 1:200, 1:400) and incubated at 37 °C for 2 h. Subsequently, an enzyme-labeled secondary antibody was added at a dilution of 1:5000 and incubated at 37 °C for 2 h. Following a final wash, 50 μL/well of 3,3′,5,5′-tetramethyl-benzidine (Solarbio, Beijing, China) substrate was added to the plates for a 15 min chromogenic reaction at room temperature in the dark. The reaction was halted by adding 50 μL/well of 2 M H_2_SO_4_ (Solarbio, Beijing, China). The optimal antigen concentration and serum dilution ratio were chosen based on the highest OD450 nm ratio of positive to negative reference serum (P/N) value and the OD450 nm of the positive serum closest to 1.0. Then, the subsequent steps of the indirect ELISA method were systematically optimized in sequence, using the highest P/N value as the criterion for evaluation. These steps included determining the optimal sequential coating conditions for streptavidin and E2EP3 peptide antigen (4 °C for 12 h, then 37 °C for 2 h; 37 °C for 2 h, then 37 °C for 2 h; 4 °C for 12 h, then 4 °C for 12 h; 37 °C for 2 h, then 4 °C for 12 h); identifying the optimal blocking solution (1% BSA, 5% BSA, 1% sodium caseinate powder, 5% sodium caseinate powder); establishing the optimal blocking time (30 min, 60 min, 90 min, 120 min); determining the optimal primary antibody incubation time (30 min, 60 min, 90 min, 120 min); selecting the optimal secondary antibody dilution (1:3000, 1:4000, 1:5000, 1:6000); ascertaining the optimal secondary antibody incubation time (30 min, 60 min, 90 min, 120 min); and defining the optimal chromogenic reaction time (5 min, 10 min, 15 min, 20 min).

#### 2.5.2. Determination of the Cut-Off Value of E2EP3-ELISA

Following the optimized reaction conditions described in Section 2.5.1, a total of 64 porcine clinical serum samples, including 32 VNT-confirmed seronegative and 32 seropositive specimens, were assayed in technical triplicate. The optical density at OD450 nm was recorded using a microplate reader. Based on the obtained photometric readings, receiver operating characteristic (ROC) curves and the corresponding area under the curve (AUC) were plotted to calculate the diagnostic sensitivity (Se) and specificity (Sp) of the established ELISA method. The optimal cut-off value was defined at the point yielding the maximum Youden index (Youden = Se + Sp − 1). Samples with an OD450 nm value greater than or equal to the threshold were judged seropositive.

#### 2.5.3. Analytic Specificity, Sensitivity, and Repeatability Evaluation of the Indirect ELISA

To assess the sensitivity of the E2EP3 ELISA, we tested twofold serial dilutions of serum ranging from 1:20 to 1:5120. To confirm the specificity of the method, we used six different porcine positive sera representing CSFV, JEV, PCV-2, PCV-3, PRV, and PRRSV. In addition, we evaluated intra-assay precision and inter-assay reproducibility by testing six replicates of GETV-positive sera on the same plate and across six independent plates at different times.

#### 2.5.4. Comparison of E2EP3-ELISA with rE2-ELISA

The coincidence rate of 82 Getah virus-positive serum samples was determined using the optimized E2EP3-ELISA and rE2-ELISA. The E2 protein was expressed in our previous study. The method was almost the same as the one described above, except for the following points: The antigen was coated onto the plates at a concentration of 10 µg/mL. The blocking time was 2 h at 37 °C.

#### 2.5.5. Detecting Clinical Swine Sera Using E2EP3-ELISA and VNT

These clinical swine sera samples (*n* = 120) were subjected to testing for GETV antibodies using the E2EP3-ELISA developed in this study, and the VNT method previously reported [25]. Virus titers were determined by plaque assay and expressed as plaque-forming units per milliliter (PFU/mL), and the neutralization titer was defined as the highest serum dilution that achieved ≥50% inhibition of virus-induced cytopathic effect (CPE), relative to the virus control group. This allowed us to evaluate the antibody-positive rate and the degree of agreement between the two techniques. Based on preliminary experimental data from our laboratory, a serum was considered positive if its neutralizing antibody titer was ≥1:4 in the VNT. The geometric mean titer (GMT) of the neutralizing antibody was computed using the titers of all positive swine sera confirmed by VNT.

#### 2.5.6. Statistical Analysis

To analyze the relationship between E2EP3-ELISA, rE2-ELISA, and VNT, we calculated CV and correlation coefficients using Excel (2605) and GraphPad Prism (8.0.2) software. The agreement is calculated as follows: Agreement = (number of true positive/negative samples/total number of positive/negative samples.

## 3. Result

### 3.1. Selection of E2EP3 as an Antigen for ELISA

**Figure 1:**
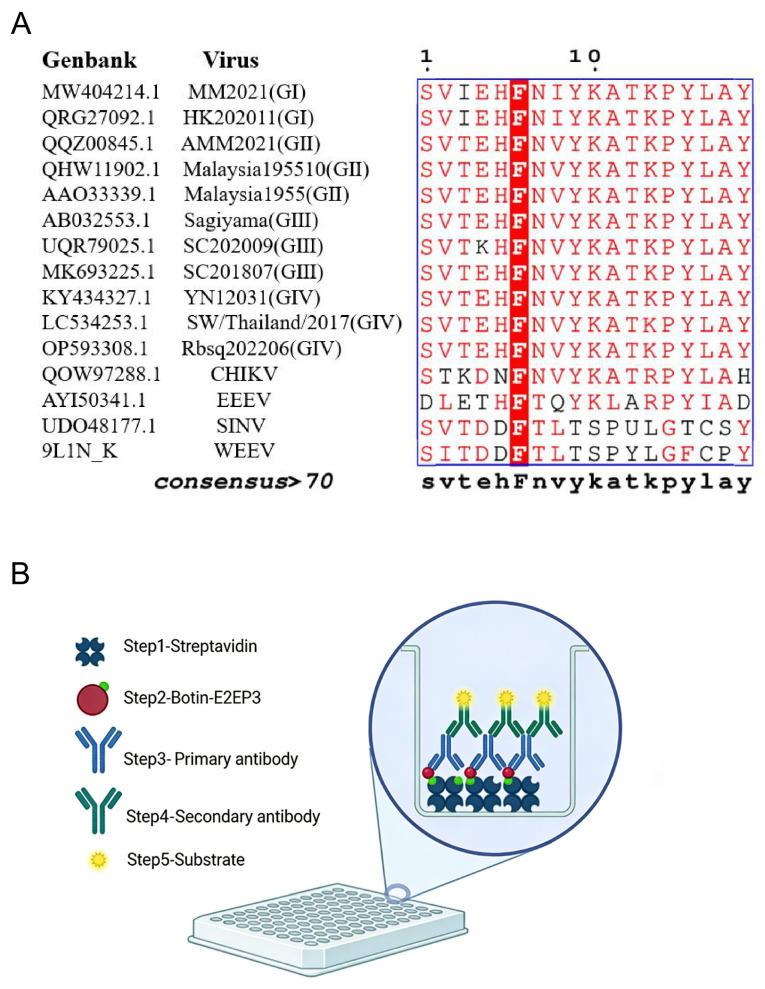
**Comparison of amino acid sequences of the E2EP3 region and schematic diagram of the indirect ELISA.** (**A**) Amino acid sequences for the E2EP3 region of the E2 protein were compared among various Getah virus strains (*n* = 11) and other alphaviruses, namely CHIKV, EEEV, SINV, and WEEV. (**B**) Microplates were pre-coated with streptavidin, followed by biotinylated polypeptide coating and blocking. Serum samples and HRP-conjugated secondary antibody were sequentially incubated, and the signal was developed with a chromogenic substrate. Created in https://BioRender.com.

Previous research identified E2EP3 as a significant early serological marker in human patients with CHIKV infection [29]. Based on this evidence, we posited that the E2EP3 epitope might play a similar role in GETV infection. We specifically hypothesized that it would effectively stimulate the production of neutralizing antibodies following GETV infection, thereby serving as a suitable target for early serological detection. To evaluate this hypothesis, we first examined the conservation of the E2EP3 sequence. GETV is classified into four distinct genotypes. Through amino acid sequence alignment, we found that the E2EP3 peptide is highly conserved among the four genotypes of GETV and also exhibited high homology with CHIKV within the alphavirus genus (Figure 1A). Based on this high degree of conservation, we selected the E2EP3 peptide as the coating antigen for developing an indirect ELISA, which completed the functional validation of the epitope based on the serological characteristics of GETV itself, further confirming the feasibility of E2EP3 as a specific diagnostic antigen.

To enhance the assay’s sensitivity, the E2EP3 peptide ELISA was established by using biotinylated E2EP3 peptide antigen attached to streptavidin-coated microplate (Figure 1B). This strategy leverages the high-affinity biotin–streptavidin interaction to amplify the detection signal and reduce non-specific background noise.

### 3.2. Detection of Antibodies by E2EP3-ELISA in Sera from Mice Experimentally Infected with GETV

**Figure 2:**
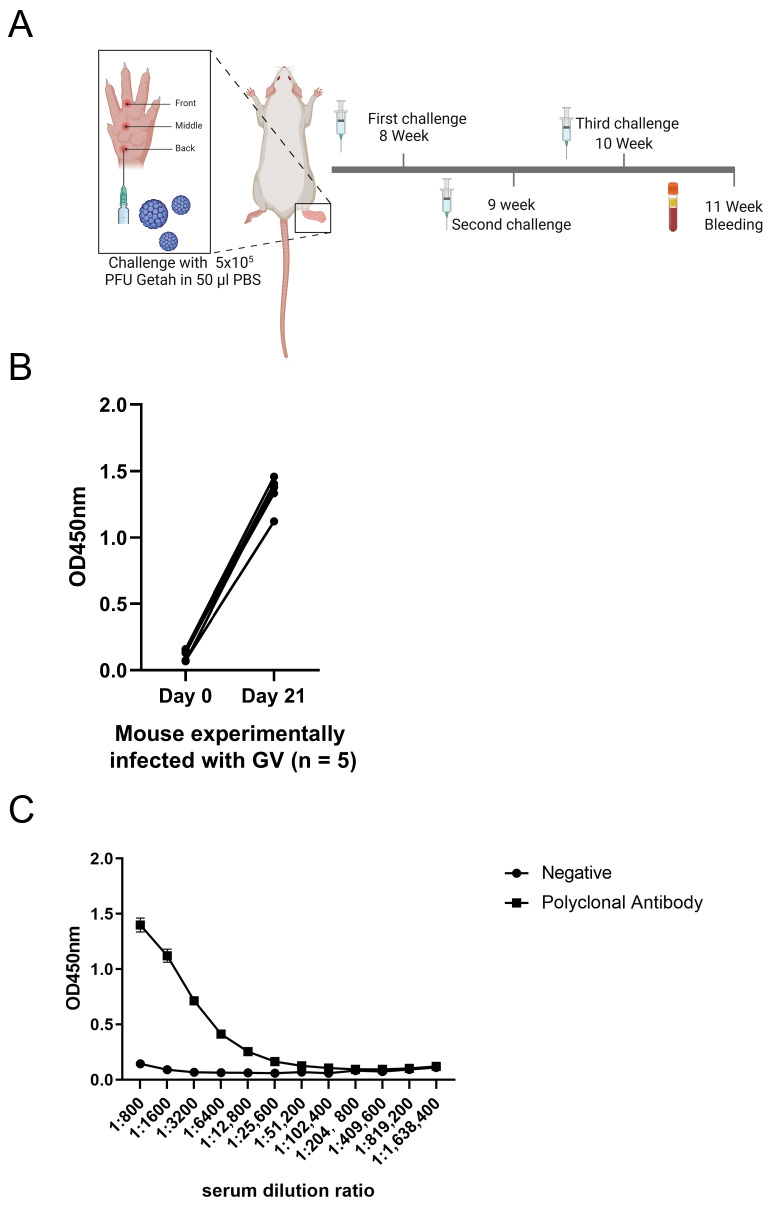
**Mouse challenge model and evaluation of Getah virus-specific antibody responses.** (**A**) Timeline of mouse footpad challenge experiments. Created in https://BioRender.com. (**B**) Application as an antigen for ELISA. E2EP3-ELISA detection of antibodies in sera from individual mice (*n* = 5) experimentally infected with Getah virus. The plate was coated with peptide E2EP3 (15 µg/mL), and the ELISA was performed as described above. Sera collected from mice at days 0 and 21 were tested at a dilution of 1:400. (**C**) Titer determination of the polyclonal antibodies against the Getah virus. Serum samples from day 0 (negative control) and day 21 (positive control) were serially diluted (1:800 to 1:1,638,400) and added to plates coated with 1 μg/well purified E2 protein. HRP-conjugated anti-mouse IgG (1:5000) was used to determine the post-challenge antibody titers.

To test the feasibility of the E2EP3-ELISA method, we challenged mice with 5 ×10^5^ PFU GETV to produce GETV-positive sera. The flow is shown in Figure 2A. Sera from mice (*n* = 5) experimentally infected with GETV were tested by E2EP3-ELISA. In the E2EP3-ELISA, serum OD values on day 0 ranged from 0.068 to 0.159 (median 0.129), and by day 21, all mice showed a significant seroconversion, with the OD450 nm values ranging from 1.160 to 1.458 (median 1.332) (Figure 2B), indicating that E2EP3-ELISA can successfully detect antibodies produced after GETV infection.

Sera from mice (*n* = 5) experimentally infected with GETV were tested by rE2-ELISA. The polyclonal antibody well OD450 nm value to negative control well OD450 nm value ratio (P/N) > 2.1 is considered positive, and the highest dilution is the titer of the polyclonal antibody. The results showed that the titer of the mouse polyclonal antibody against the GETV whole virus reached 1:25,600 (Figure 2C).

### 3.3. Screening of Optimal Reaction Conditions for Indirect ELISA

**Figure 3:**
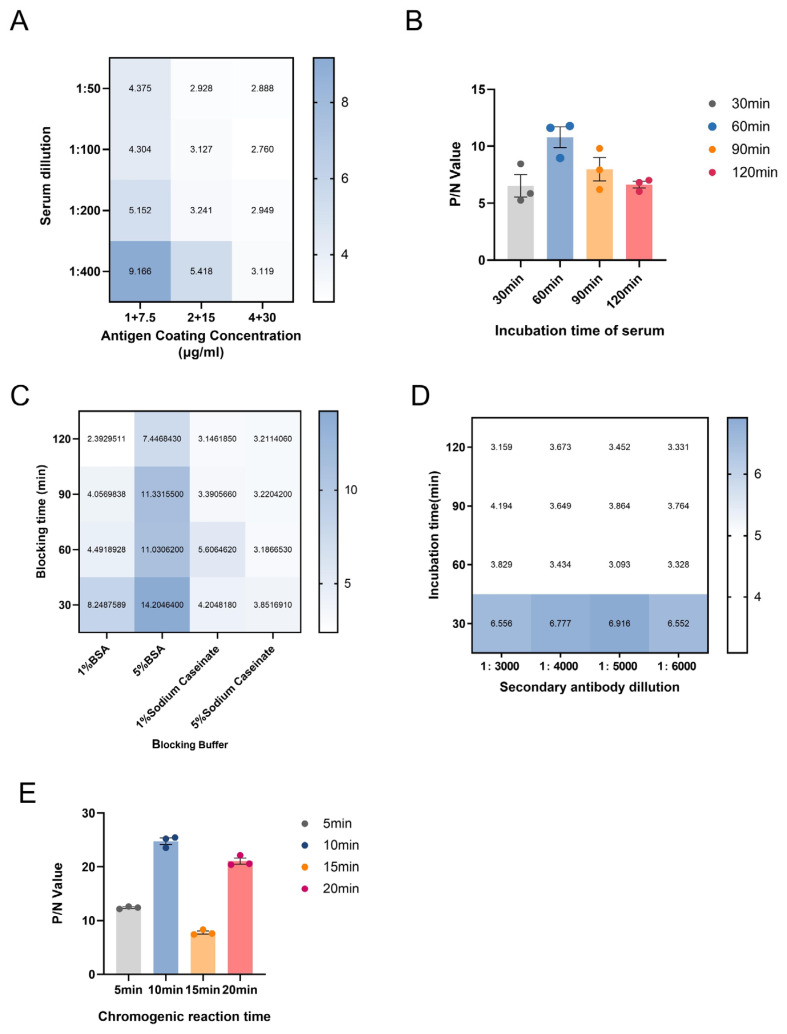
**Optimization of E2EP3-ELISA working conditions.** (**A**) Optimization of the concentration of coating antigen and serum sample dilution; (**B**) serum incubation time; (**C**) blocking buffer; (**D**) secondary antibody (HRP-conjugated goat anti-mouse IgG secondary antibody) dilution; (**E**) chromogenic reaction time. The reaction conditions were considered optimal when the OD450 ratio between the positive and negative serum (P/N value) reached the highest, and the OD450 nm value of positive serum was close to 1.0, whereas that of negative serum was as low as possible.

The optimal ELISA conditions were established by checkerboard titration. The coating concentration of streptavidin and E2EP3 was optimized to 1 μg/mL and 7.5 μg/mL, while the optimal dilutions for serum and the secondary antibody were 1:400 and 1:5000, respectively. A 5% BSA blocking buffer applied for 30 min was most effective. Furthermore, the reaction conditions of E2EP3-ELISA were optimal when the reaction times of streptavidin, E2EP3, serum, secondary antibody, and substrate were 120, 120, 60, 30, and 10 min, respectively (Figure 3C).

### 3.4. Calculation of Cut-off Value for E2EP3-ELISA

**Figure 4:**
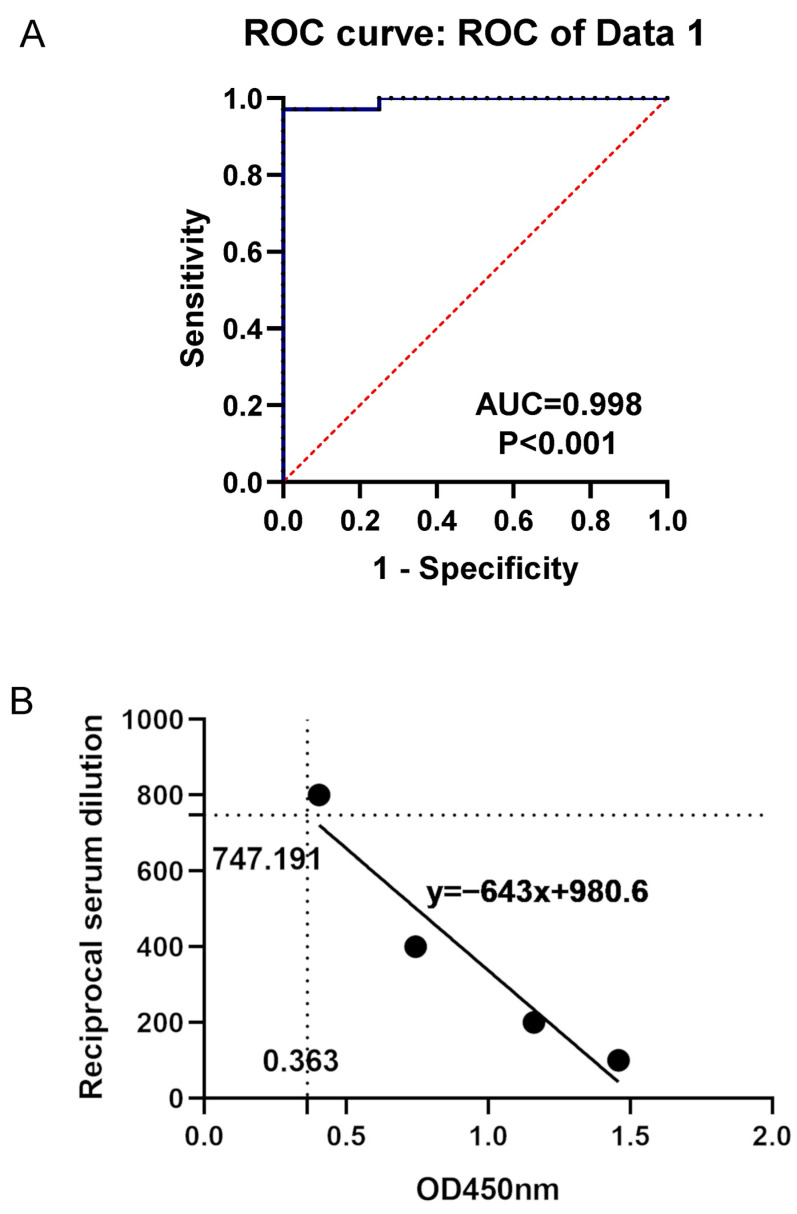
**Determination of cut-off value.** (**A**) The cut-off value was statistically calculated using 32 porcine negative sera to determine the threshold between negative and positive results. (**B**) Calculation of a standard serum dilution equivalent to the tentative cut-off value in the E2EP3-ELISA. Serial dilutions of a standard serum (day 21 of mice) from 1:100 to 1:800 were tested using the E2EP3-ELISA. The tentative cut-off value calculated from the negative control sera (0.363) was equivalent to that given by the standard serum diluted to 1:747.2.

To define the threshold value of the established E2EP3-ELISA assay, a total of 32 VNT-validated seronegative and 32 seropositive porcine serum samples were subjected to detection with this assay. ROC curve analysis (Figure 4) revealed an AUC value of 0.998, with a 95% confidence interval of 0.9778 to 1.000 and a statistical P value of <0.0001. The maximum Youden index was obtained at a true positive rate of 0.9714 and a true negative rate of 1.0000. Correspondingly, the optimal cut-off value was determined as 0.363. Specimens with an OD450 nm reading ≥0.363 were judged to be GETV seropositive.

**Figure 5:**
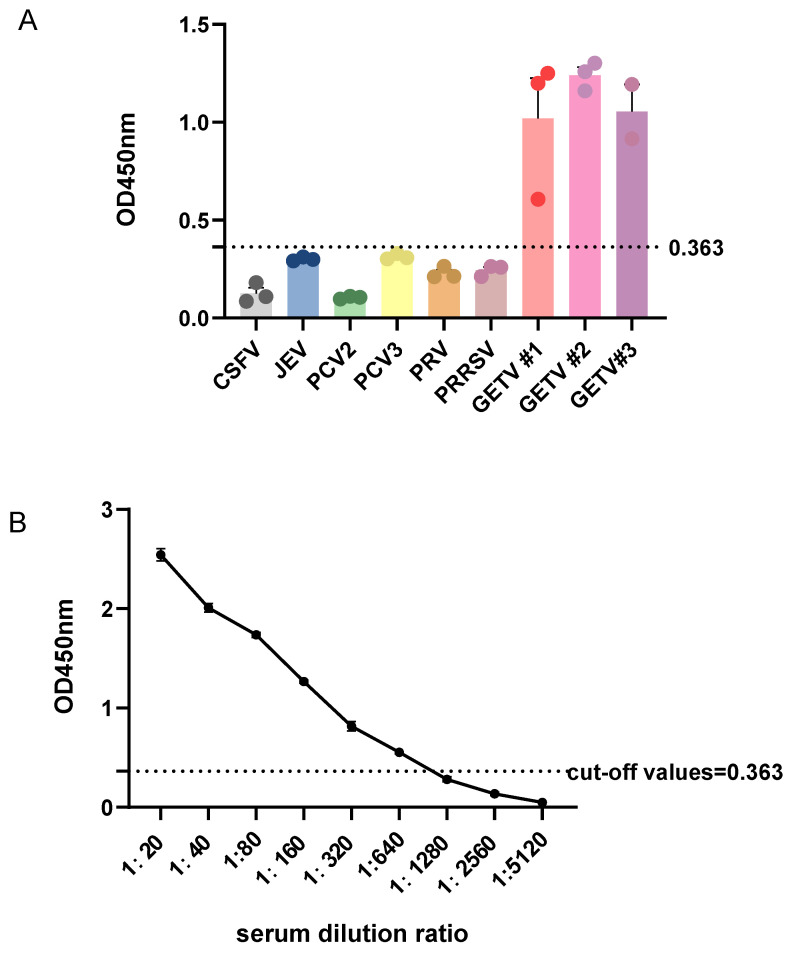
**Specificity and sensitivity of E2EP3-ELISA.** (**A**) Swine positive sera against PCV2, PCV3, CSFV, JEV, PRV, and PRRSV, and GETV positive serum were tested using the E2EP3-ELISA, and the mean OD450 value was calculated to determine the positivity of the sample. The dashed line indicates the cut-off value (0.363) of E2EP3-ELISA. (**B**) Nine dilutions of GETV-positive serum were used to test the sensitivity of E2EP3-ELISA. The dashed line indicates the cut-off value (0.363) of E2EP3-ELISA.

To approximately convert the cut-off value into a standard serum dilution, serial two-fold dilutions of the standard serum were tested in the same batch (Figure 4B). According to the standard curve equation (y = −643x + 980.6), an OD value of 0.363 corresponds to a standard serum dilution of 1:747.191, which is close to the critical dilution in the subsequent ELISA sensitivity assay (Figure 5B).

### 3.5. Specificity, Sensitivity, and Repeatability of the E2EP3-ELISA

To evaluate the specificity of the E2EP3-ELISA, the assay was tested using three GETV-positive swine serum samples: two obtained from naturally infected pigs in different provinces, and one collected from a pig immunized with a GETV vaccine on our farm. The assay was also tested against positive sera for six other porcine pathogens (PCV2, PCV3, CSFV, JEV, PRV, and PRRSV). The OD450 nm values for the GETV-positive sera ranged from 0.916 to 1.320. In contrast, the OD450 nm values for sera containing antibodies against the non-target pathogens fell between 0.0967 and 0.329, all below the established cut-off value of 0.363 (Figure 5A). These findings demonstrate that the E2EP3-ELISA exhibits high specificity for GETV antibodies and shows no cross-reactivity with antibodies against the other pathogens.

To assess the sensitivity of the E2EP3-ELISA, a GETV-positive swine serum sample was subjected to serial dilution. The result showed that even at a dilution of 1:640 (consistent with the standard curve conversion results in Figure 4B), the OD450 nm value remained above the established cut-off of 0.363, indicating that the sample was still classified as positive (Figure 5B). This demonstrates that the developed E2EP3-ELISA exhibits high sensitivity and is capable of detecting low levels of GETV-specific antibodies in serum samples.

To evaluate the repeatability of the E2EP3-ELISA, six GETV-positive serum samples were analyzed, each tested with three replicates, with consistent serum sample sources and the same batch of coated microplates. The coefficients of variation (CV) obtained were 1.04–4.76% (intra-assay) and 0.22–7.24% (inter-assay). All CV values were below the widely accepted threshold of 10%, confirming the high consistency and reproducibility of the assay (Table 1).

### 3.6. Comparative Analysis of E2EP3-ELISA and E2-ELISA for Detection of GETV Antibodies in Clinical Porcine Serum Samples

To directly compare the E2EP3-ELISA with the established E2-ELISA, 82 well-characterized clinical porcine serum samples (*n* = 82) were tested in parallel using both assays. Both methods yielded identical results, identifying 50 positive and 32 negative samples without any classification discrepancies (Table 2). This perfect agreement was statistically confirmed by Cohen’s kappa coefficient analysis, which produced a kappa value of 1.00 (95% CI: 1.000–1.000), with a statistical significance of P < 0.001. The results demonstrate that the newly developed E2EP3-ELISA performs equivalently to the E2-ELISA for detecting GETV antibodies in clinical samples.

### 3.7. Comparison of E2EP3-ELISA with VNT

Using the E2EP3 peptide-based indirect ELISA developed in this study and the virus neutralization test (VNT) in parallel, we detected GETV antibodies in clinical porcine serum samples (*n* = 120; 6 of which were from vaccinated pigs). The results showed that the ELISA identified 88 positive samples and 32 negative samples, while the VNT identified 82 positive samples and 38 negative samples. Using the VNT results as the reference standard, the positive percent agreement of the E2EP3 peptide-based indirect ELISA was 100%, the negative percent agreement was 84.2%, and the overall percent agreement was 95% (Table 3). The relatively low negative percent agreement of 84.2% is attributed to the higher sensitivity of the ELISA, which can detect some weakly positive sera that are not identified by VNT (Figure S2). Overall, the ELISA demonstrates higher sensitivity. This performance highlights a key advantage of the E2EP3-ELISA: its applicability for reliable detection even in cases where neutralizing antibody titers are relatively low.

## 4. Discussion

In recent years, GETV has emerged as an important mosquito-borne pathogen, with multiple outbreaks reported on pig farms in several provinces of China, including Hubei, Sichuan, Yunnan, Hainan, Henan, and Guangdong [9,35]. The increasing number of outbreaks and the emergence of variant strains highlight the growing threat posed by GETV to the swine industry and potentially to public health [36]. However, the lack of efficient and commercially available diagnostic tools for detecting GETV antibodies in pigs remains a major obstacle to large-scale seroepidemiological surveillance and effective disease control.

Among the currently available diagnostic methods, ELISA is widely used because of its high sensitivity, specificity, and suitability for large-scale screening. Existing GETV ELISA assays mainly employ two types of antigens. The first approach uses purified GETV viral particles as antigens [37]; however, this method raises concerns regarding biosafety and antigen consistency. By contrast, most current assays rely on recombinant E2 protein or partial E2-derived peptides. The E2 protein is highly conserved and strongly immunogenic, and it plays a critical role in inducing neutralizing antibodies during infection, making it a common target for serological diagnosis. Nevertheless, the production of recombinant E2 protein often involves expression in *Escherichia coli*, in which the protein is frequently produced as inclusion bodies, leading to complex purification procedures, longer production cycles, and potential batch-to-batch variability.

In the present study, we developed an indirect ELISA based on the synthetic peptide E2EP3 for the detection of GETV-specific IgG antibodies in swine serum. E2EP3 is located within the N-terminal region of the GETV E2 protein and represents a conserved immunodominant epitope. By synthesizing a biotinylated E2EP3 peptide and utilizing the strong affinity interaction between biotin and streptavidin for signal amplification, the assay achieved enhanced antibody detection sensitivity. The use of the biotin–streptavidin amplification system likely contributed to the enhanced signal intensity and reduced background noise observed in this assay [38]. Compared with recombinant protein-based ELISAs, the peptide-based strategy offers several advantages, including high antigen purity, consistent quality between batches, a shorter production cycle, and reduced manufacturing cost, which may facilitate large-scale diagnostic applications.

After optimization of the assay conditions using square array titration, an appropriate cut-off value for the E2EP3-based ELISA was established. Evaluation using clinical porcine serum samples demonstrated that the assay could clearly discriminate GETV-positive from negative samples, exhibiting high specificity, sensitivity, and reproducibility. In the cut-off value determination assay, the OD450 nm values of tested clinical serum samples showed nearly no overlap between groups, with no cross-boundary samples. Accordingly, the ROC curve exhibited a typical right-angle stepwise pattern with very few intermediate inflection points, further verifying the high sensitivity and specificity of the established ELISA method. In addition, the ability of the assay to detect antibodies in sera diluted up to 1:640 further supports its high analytical sensitivity compared with conventional ELISA formats. These findings indicate that the E2EP3-based ELISA represents a reliable method for serological detection of GETV infection in pigs. GETV infection has also been reported in other susceptible animal species, particularly horses [39]. In the present study, only porcine serum samples were evaluated; therefore, the performance of this assay in detecting antibodies from other host species remains to be determined. Future studies should assess the applicability of this method using sera from additional susceptible animals in order to determine its broader utility for multi-species surveillance of GETV infection. In addition, the number and geographic distribution of clinical samples included in this study were relatively limited, and further validation using larger sample sets from different regions will be necessary to fully evaluate the robustness of the assay.

Interestingly, previous studies on CHIKV, another member of the genus *Alphavirus*, have shown that antibodies targeting the E2EP3 epitope are generated during the early phase of infection and that IgG3 is the predominant antibody subtype recognizing this epitope [31]. Given the sequence conservation of the E2EP3 region among alphaviruses, it is possible that a similar immune recognition pattern may occur during GETV infection. This raises the possibility that antibodies against the E2EP3 epitope could serve as early serological markers of GETV infection. Nevertheless, it should be noted that the E2EP3 epitope was initially based on referencing conserved epitope regions of CHIKV. Importantly, its specific immunoreactivity was further validated using serum samples from natural GETV-infected pigs. Thus, the selection of E2EP3 is supported by GETV experimental evidence rather than relying solely on extrapolation from other alphaviruses. However, the specific antibody subclasses induced by GETV infection in pigs and their kinetics during infection have not yet been fully characterized. In addition, the relationship between the appearance of anti-E2EP3 antibodies and the dynamics of viremia following GETV infection remains unclear and warrants further investigation.

This study presents several inherent limitations. Clinical serum samples were confined to a narrow geographical range, potentially failing to fully represent the serological profiles of GETV strains circulating across distinct endemic areas. The pool of GETV-positive specimens utilized for methodological validation was relatively constrained, with limited inclusion of independent positive sera from heterogeneous origins; this insufficient sampling may not adequately capture inter-individual biological variation and could introduce modest evaluation bias. Cross-reactivity with other alphaviruses like CHIKV also remains to be systematically explored in further investigations. Subsequent efforts will broaden both sample scale and geographical coverage, while incorporating additional independent field-derived positive sera for corroborative validation. Such improvements will further strengthen the practical applicability and detection reliability of the developed ELISA assay.

In summary, we developed a peptide-based ELISA targeting the E2EP3 epitope for the detection of GETV antibodies. The assay demonstrated good sensitivity, specificity, and reproducibility when evaluated using clinical porcine serum samples, and showed high agreement with the serum neutralization test. These results suggest that the E2EP3-based ELISA represents a promising tool for serological surveillance and diagnosis of GETV infection and may provide a useful foundation for the development of commercial diagnostic kits for GETV.

## Figures and Tables

**Table 1:** Repeatability of the E2EP3-ELISA.

Repeat	Sample												
		1		2		3		4		5		6	
Batch		Average	CV(%)	Average	CV(%)	Average	CV(%)	Average	CV(%)	Average	CV(%)	Average	CV(%)
Intra	1	2.443	2.26%	1.621	4.76%	1.373	1.04%	2.861	2.09%	2.398	3.18%	1.995	3.16%
	2	2.347		1.474		1.359		2.970		2.257		2.110	
	3	2.352		1.565		1.387		2.870		2.368		2.003	
Inter		2.471	2.64%	1.558	0.22%	1.468	4.71%	2.770	3.26%	2.399	1.72%	2.256	7.24%

**Table 2:** The test results for 82 pig serum samples by E2-ELISA and E2EP3-ELISA.

Coating Antigen	Serotype	Positive	Negative	Positive Rate
E2	Immune Vaccine Serum	6	0	100%
	Clinical Positive Serum	44	0	100%
	Negative Serum	0	32	0%
E2EP3	Immune Vaccine Serum	6	0	100%
	Clinical Positive Serum	44	0	100%
	Negative Serum	0	32	0%

**Table 3:** The test results for 120 pig serum samples by VNT and E2EP3-ELISA.

		VNT	
	Positive	Negative	Total
ELISA Positive	82	6	88
ELISA Negative	0	32	32
Total	82	38	120
Agreement	100%	84.2%	95%

## Data Availability

The original contributions presented in this study are included in the Supplementary Material. Further inquiries can be directed to the corresponding author.

## Supplementary Materials

The following supporting information can be downloaded at: https://www.mdpi.com/article/10.3390/vetsci13060530/s1, Table S1: Detailed information of strains in sequence alignment; Figure S1: OD450nm Data of Clinical Sera for ROC Curve Plotting; Figure S2: Comparison of Sensitivity between ELISA and VNT; Figure S3: Individual antibody levels of experimental mice for polyclonal serum preparation.
