# Supplementary material for: Establishment and Application of an Indirect ELISA for Detecting Getah Virus IgG Antibodies in Swine Based on the E2EP3 Peptide
Source: Vet Sci. 2026 May 29;13(6):530. doi: 10.3390/vetsci13060530 (PMC13308481; doi:10.3390/vetsci13060530)

Supplementary information

Table S1:Detailed information of strains in sequence alignment

Genbank	Virus	Sources
MW404214.1	MM2021(GI)	Malaysia
QRG27092.1	HK202011(GI)	Hong Kong, China
QQZ00845.1	AMM2021(GII)	Malaysia
QHW11902.1	Malaysial95510(GII)	Malaysia
AA033339.1	Malaysia1955(GII)	Malaysia
AB032553.1	Sagiyama(GII)	Japan
UQR79025.1	SC202009(GII)	Sichuan, China
MK693225.1	SC201807(GII)	Sichuan, China
KY434327.1	YN12031(GIV)	Yunnan, China
LC534253.1	SW/Thailand/2017(GIV)	Thailand
OP593308.1	Rbsq202206(GIV)	China
QOW97288.1	CHIKV	Reference strain
AYI50341.1	EEEV	Reference strain
UDO48177.1	SINV	Reference strain
9LIN_K	WEEV	Reference strain

Figure S1: OD450nm Data of Clinical Sera for ROC Curve Plotting

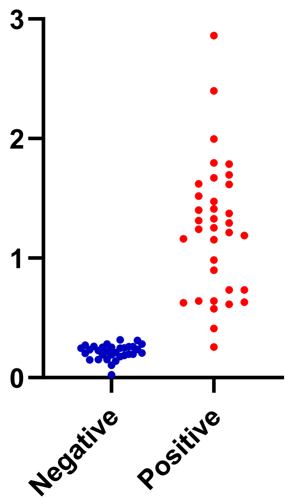

Figure S2: Comparison of Sensitivity between ELISA and VNT.

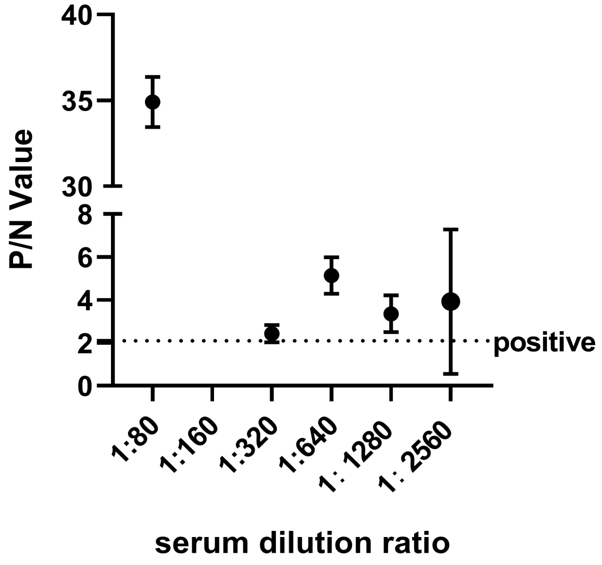

Number	Serum Type	VNT Endpoint Titer	P value OD450nm	N value OD450nm	P/N Value
1	Porcine serum	1:80	2.363	0.068	34.877
2	Porcine serum	1:1280	0.210	0.076	2.779
3	Porcine serum	1:1280	0.224	0.076	2.962
4	Porcine serum	1:1280	0.239	0.076	3.042
5	Porcine serum	1:1280	0.350	0.076	4.624
6	Porcine serum	1:640	0.376	0.076	4.968
7	Porcine serum	1:1280	0.350	0.076	4.624
8	Porcine serum	1:1280	0.210	0.076	2.780
10	Porcine serum	1:640	0.453	0.076	5.992
11	Porcine serum	1:640	0.267	0.076	3.524
12	Porcine serum	1:320	0.165	0.080	2.069
13	Porcine serum	1:1280	0.633	0.076	8.380
14	Porcine serum	1:2560	0.105	0.079	1.314

Figure S3: Individual antibody levels of experimental mice for polyclonal serum preparation

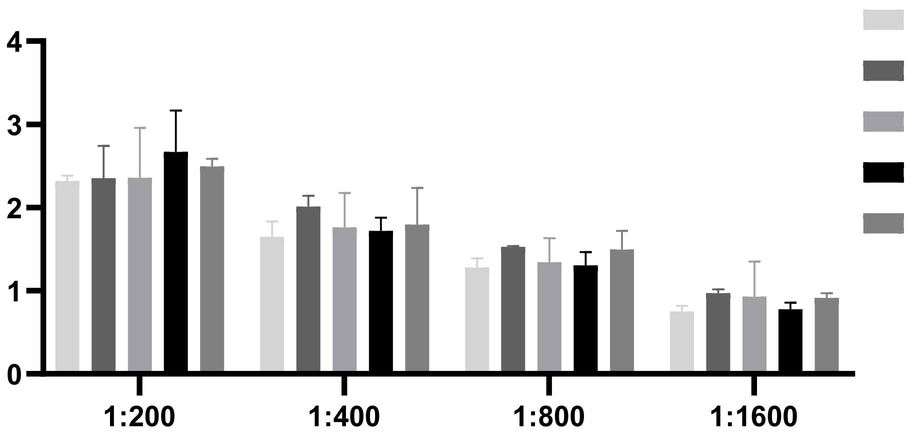

Supplement: Supplementary file 1 [file vetsci-13-00530-s001.zip › vetsci-4289214-supplementary.pdf]
